# A Pilot Study of Functional Brain Magnetic Resonance Imaging in BPS/IC Patients: Evidence of Central Sensitization

**DOI:** 10.5152/tud.2024.23209

**Published:** 2024-01-01

**Authors:** Pedro Abreu-Mendes, Diogo Dias, Francisca Magno, Guilherme Silva, José Rodrigues-Fonseca, Paulo Dinis, Francisco Cruz, Rui Almeida Pinto

**Affiliations:** 1 Department of Urology, Centro Hospitalar e Universitário de São João, Porto, Portugal; 2 Department of Surgery and Physiology, University of Porto, Faculty of Medicine, Porto, Portugal; 3 Institute for Research and Innovation in Health (i3S), University of Porto, Porto, Portugal; 4 Department of Gynecology and Obstetrics, Maternidade Alfredo da Costa, Lisbon, Portugal; 5 Department of Neuroradiology, Centro Hospitalar Universitário de São João, Porto, Portugal

**Keywords:** Interstitial cystitis, bladder pain syndrome, brain magnetic resonance imaging, neuroimaging, central sensitization

## Abstract

**Objective:**

Bladder pain syndrome/Interstitial cystitis (BPS/IC) is characterized by increased activity in bladder afferent pathways, recruitment of silent nociceptive neurons, and sensitization of the brain areas responsible for pain amplification. Default mode network (DMN) is a set of regions activated during the resting state, which reflect the brain’s intrinsic activity. Conversely, the sensorimotor network (SMN) plays a key role in structural neuroplasticity. This study aimed to evaluate DMN and SMN activity in BPS/IC patients, both with and without bladder noxious stimulus, using functional brain magnetic resonance imaging (MRI).

**Methods:**

Six BPS/IC female patients underwent 3 Tesla fMRI brain scanners. Acquisitions consisted of 10-minute blood oxygen level-dependent echo-planar imaging. The first acquisition was with an empty bladder, painless, and the second was with suprapubic pain. Data were processed using the independent component analysis method with the MELODIC tool from the functional brain MRI of the Brain Software Library (FSL). A semi-quantitative analysis was performed afterward.

**Results:**

The patients’ age was 42.6 ± 5 years, pain intensity was 7 ± 0.7 (0-10), day and night frequency were 9.2 ± 2.2 and 2.8 ± 1.0, and maximal bladder capacity was 260 ± 54 mL. One patient was unable to complete the study. All patients showed a comparable DMN activation in both empty and full bladder states, and all presented high SMN activation whether the bladder was empty or full.

**Conclusion:**

The activation of DMN at both bladder states, empty and full, and constant SMN activation without and with pain supports the role of these networks in BPS/IC. Similar findings have been reported in other chronic pain syndromes.

**Main Points**

- Central sensitization plays a role in the BPS/IC pathophysiology.
- Both central nervous system pathways, default mode network, and sensorimotor network seem to be involved in BPS/IC pathophysiology.
- In the future, functional neuroimaging might be a useful tool in the approach of BPS/IC.

## Introduction

Refractory bladder pain syndrome/Interstitial cystitis (BPS/IC) is a chronic pain condition characterized by suprapubic pain or discomfort, along with lower urinary tract symptoms such as urinary frequency, urgency, or nocturia, without any identifiable infection.^1^ This disease, which mainly affects active women at fertile age, is a massive burden to health-care services. Its incidence is probably underestimated, despite a recent study suggesting that almost 9 in 10 women with pelvic pain and urinary complaints suffer from BPS/IC.^2,3^ Significantly worst quality of life (QoL), anxiety, depression, and sexual dysfunction are frequently reported.^1^

A complete understanding of the pathophysiology of this condition is imperative to tailor available therapeutic options appropriately. Growing evidence points to a multifactorial etiology for BPS/IC: urothelial dysfunction, neurogenic bladder inflammation, and mast cell activation seem to be involved.^4^ Increasing levels of urothelial-released substances (adenosine triphosphate and nitric oxide), inflammatory agents (prostaglandin E2, histamine, adenosine, and serotonin), and neurotrophic agents can lead to functional changes in bladder afferent pathways, especially through changes in C-fibers. These fibers, when activated, are responsible for the slow transmission of pain, transmitting afferently noxious inputs to the dorsal horn neurons in the spinal cord.^5^ When afferent hypersensitivity to noxious stimuli exists, there is increased stimulation of the central nociceptive pathways.^6^ In fact, a continuous nociceptive peripheral stimulus can lead to a decrease in the threshold for the activation of spinal cord neurons, a phenomenon closed linked to allodynia. This continuous activation of a central pain pathway increases the number of neurons in this pathway (neuroplasticity events), activating specific brain areas and amplifying pain, which explains a central sensitization for pain.^6,7^ This neuronal upregulation of pain can explain the reason for nonnoxious normal stimuli, like a mild bladder filling, causing pain.^7^ Central pain amplification plays a leading role in BPS/IC pathophysiology, as confirmed by previous studies.^5^

Advanced neuroimaging techniques made the study of pain pathways possible, including the brain response to noxious stimuli.^8^

Functional brain magnetic resonance imaging (MRI), a method with spatial resolution, locates activated areas of the brain through blood oxygen level-dependent (BOLD) contrast images.^9^ Resting-state functional brain MRI is a task-independent and noninvasive technique that different methods can process. One of the most frequently used is the independent component analysis, which decomposes data in different brain networks. The brain divides itself into networks. Each network is a group of brain areas that are structurally and functionally linked, sharing a common purpose.

For example, some networks already described are the default mode network (DMN), the sensorimotor network (SMN), the basal ganglia, the visual network, the visuospatial network, the salience network (SN), the language network, and the precuneus network, among others.^10,11^ Neuroimaging technique allows the study of the intrinsic activation of the brain without stimulus.^12^ However, to compare the effect of painful stimuli on the activity of certain brain structure, images should be acquired with nonnoxious input and then with noxious stimuli, such as the pain and discomfort perceived by a full bladder.^13^

Pain is characterized by its sensory-discriminative properties and cognitive, emotional, motivational, and affection mechanisms.^14^ Despite not being fully comprehended, several brain areas are thought to be involved in a “pain processing and integrating network” and are consistently activated across all studies.^15^

Data suggest the role of the disruption of the DMN and SN in chronic pain patients, along with alterations in the SMN.^16^ Default mode network is a set of regions activated during rest and suspended during specific tasks, reflecting the brain’s intrinsic activity. This network includes the medial prefrontal cortex, posterior cingulate cortex, and inferior parietal cortex and precuneus. Patients with chronic pain revealed a disruption in DMN, failing to show typical tasks or pain-related DMN deactivation.^16,17^ The SN is responsible for the dynamic of other networks and is involved in controlling cognition processes.^18^ It comprises the dorsal anterior cingulate cortex, bilateral insula, and pre-supplementary motor area. A recent data study suggests that higher activity in SN leads to increased functional connectivity between the DMN and executive network and within the DMN, proposing that SN may modulate the DMN.^18^ In fact, Hemington et al previously suggested that alterations in cross-network connectivity can be the key to understanding pain phenomenon in chronic conditions, as previously described for other neurological disorders.^16^ Another network, the SNM, covers the primary sensory and motor areas of the brain. Activation of SMN in the resting state has been documented in other chronic pain conditions, as related in the latest studies on functional and structural neuroplasticity, and seems to be related to sympathetic nervous system overactivity.^19^

Our study aimed to evaluate DMN, SN, and SMN patterns of activation and deactivation in BPS/IC patients, by functional brain MRI images, comparing these networks with and without noxious stimuli (empty and full bladder status, respectively).

## Material and Methods

The enrolled BPS/IC patients were informed about the study protocol, procedures, possible complications, and signed informed consent to participate in this study. Ethics Committee of Centro Hospitalar e Universitário de São João approved this pilot study (Protocol number: 284/17).

Six women with BPS/IC, diagnosed by the European Society for the Study of Interstitial Cystitis (ESSIC) criteria, were enrolled.^1^ Patients were evaluated by physical examination, O’Leary-Sant score for symptoms and problems (OSS), the pain intensity score in a 10-point visual analog scale (VAS), completed a 3-day voiding chart to evaluate the urinary frequency and maximum voided volume, question number 6 from International Prostate Symptom Score for quality of life, and the ESSIC classification for the presence of Hunner’s lesions.^20^

Afterward, patients underwent functional brain MRI imaging, acquired on a 3 Tesla MRI scanner, closed bore – Siemens Magnetom Trio, with a gradient amplitude of 45 mT/m, maximum slew rates of 200 mT/m/ms, and frequency range of 127 MHz.

Structural images were obtained using a magnetization-prepared rapid gradient-echo sequence with the following parameters: flip angle = 9°, repetition time (TR) = 2100 ms, echo time (TE) = 3.38 ms, slice thickness = 1.09 mm, 160 slices, and a voxel size of 1.1 × 1.1 × 1.1 mm.

The image acquisition procedure, including bladder catheterization, was explained to patients before entering the examination room and during the procedures. Subjects voided before entering the scanner room. The acquisitions consisted of a 10-minute BOLD imaging at rest with an empty bladder, with no referred suprapubic discomfort (empty bladder status). After the first scan, a 14-French bladder catheter was introduced, then it was infused with saline solution (NaCl, 0.9%) at room temperature until reaching the bladder’s maximum capacities, previously obtained for each patient by 3-day voiding charts. A second scan was then acquired with the bladder at maximum capacity, associated with suprapubic discomfort (full bladder status).

Data were processed based on the independent component analysis method using the MELODIC tool from the Functional magnetic resonance imaging of the Brain Software Library (FSL).

An experienced neuroradiologist evaluated the final components. The DMN, SMN, and SN were qualitatively compared between the two acquisitions (in each state) for each patient.

## Results

Demographic data and patient baseline characteristics, including age, gender, the pain score in VAS, ESSIC criteria, OSS, QoL, and bladder maximum capacities, are shown in Table 1.

The mean age of patients was 42.6 ± 5 years, the pain intensity in the VAS was 7.0 ± 0.7, OSS was 24.8 ± 1.9, day and night frequencies were 9.2 ± 2.2 and 2.8 ± 1.0, respectively; maximal bladder capacity was 260 ± 54 mL and QoL score (0-6) was 5 ± 1. Four patients presented Hunner’s lesions in cystoscopy. One of the patients was unable to finish the fMRI procedure.

**Figure 1.**
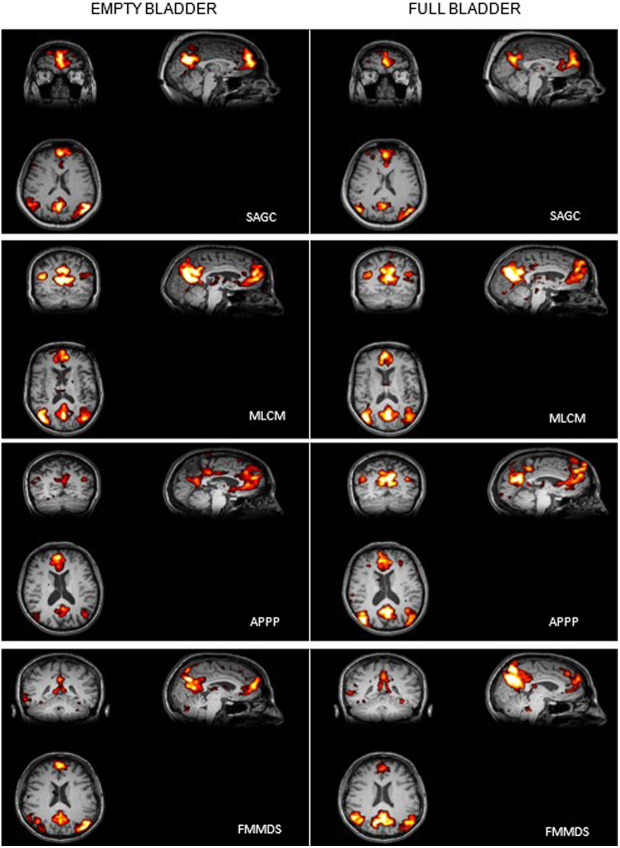
Default mode network comparisons examples. DMN empty bladder (left) compared with DMN full bladder (right) from SAGC, MLCM, APPP, and FMMDS patients.

**Figure 2.**
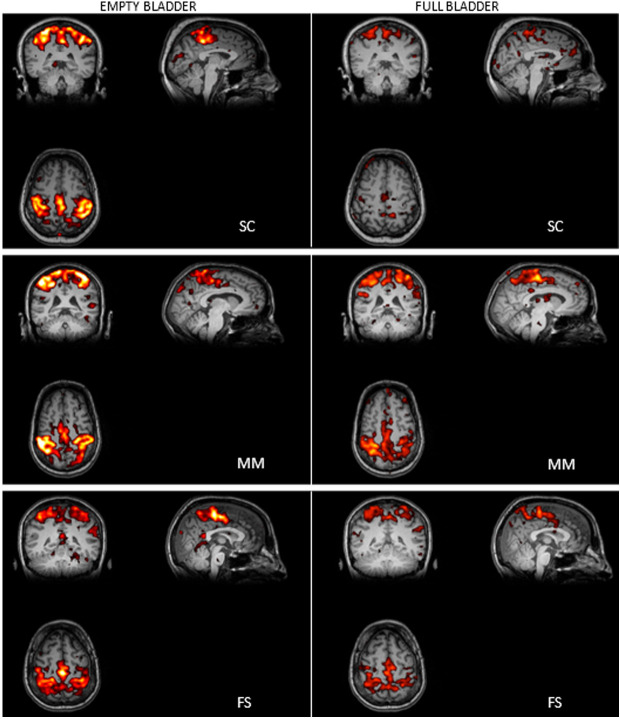
Sensorimotor network comparisons examples. SMN empty bladder (left) compared with SMN full bladder (right) from SAGC, MLCM, and FMMDS patients.

Functional brain MRI consistently evaluated both DMN and SN activations. Sensorimotor network was impossible to assess by this technique. The results are described in Table 2. Functional brain MRI images, processed by MELODIC software, are illustrated in Figures 1 and 2 as an example of the obtained and evaluated images.

All patients showed comparable DMN activation at empty and full bladder status. All of them presented higher SMN activation at an empty bladder status. Given these findings, no correlation was seen between the phenotype with Hunner’s lesions.

One of the patients, APPP, with known facial paralysis, had unexpected results in SMN. This patient presented independent networks for the face, right hand, left hand, and lower limbs, and it was impossible to detect SMN at a full bladder status to compare it with the correspondent SMN at an empty bladder status.

## Discussion

The results from our pilot study showed comparable DMN activation at empty and full bladder status, corroborating the hypothesis of DMN disruption in chronic conditions through alterations in cross-network connectivity.^16^ This imbalance may be the underlying mechanism observed in the DMN dysfunction presented in our BPS/IC women, as previously described in chronic pain patients and other mental disorders, like schizophrenia.^12^

Present data support the involvement of DMN and SMN in chronic pain syndromes, as reported before.^16^ Consistent and overlapping DMN activation at both empty and full bladder conditions could explain a “default mode of bladder pain” at an empty bladder status, a phenomenon previously described as central sensitization, which has a significant role in the pathophysiology of BPS/IC. Moreover, the absent deactivation of DMN in a full bladder state (with noxious stimulus) matches the hypothesis that chronic pain can reorganize the dynamics of DMN, that may underlie some of the attentional and cognitive changes that appear with pain.^17^

Changes in brain connectivity, gray matter volume (structure), and white matter abnormalities (information flow) have been explicitly identified in BPS/IC pathology.^21,22,23^ White matter is responsible for neural transmission in the brain, and abnormalities in these tracts implicate variations in axonal integrity, myelination, fiber organization, or axonal bifurcating that can cause disturbances in brain connectivity.^24^ Bladder pain syndrome/Interstitial cystitis shows, like other chronic pain conditions, irregularities in white matter anisotropy, which can be related to the severity of urinary symptoms, pain, and impaired QoL.^23^ Increased anisotropy can hypothetically result from the formation of de novo neural axons associated with central sensitization, “an amplification of neural signaling within the CNS that elicits pain hypersensitivity,” as previously described in BPS/IC.^7^ Kilpatrick et al, studying intrinsic oscillations and connectivity in brain activity of BPS/IC patients, found a reduced low-frequency power in the posterior insula and increased connectivity in sensorimotor cortices.^11^ These authors also found increased functional connectivity to the midbrain and cerebellum during bladder filling (pain status).^21^ Kairys et al showed increased brain gray matter volume in BPS/IC patients in the right primary somatosensory cortex, superior parietal lobule/precuneus, and right SMA. The precuneus is included in DMN, and the authors speculated that increased volumes in this area could also be related to connectivity disturbances between pain networks and DMN, as described in fibromyalgia.^22^ In brief, disturbances in brain connectivity support the predisposition for cross-connectivity in these chronic pathologies.

As previously reported, we expected to obtain an increased signal in SMN full bladder compared to empty bladder status. However, our results were divergent. A recent article related autonomic brain network activation to the sensorimotor cortices, namely the dorsal attention stream, medial prefrontal cortex, and visual cortex.^24^ The adrenergic system was previously implicated in BPS/IC pathophysiology through sympathetic nervous system overactivity.^25^ We evaluated SMN and consistently obtained higher SMN activation at baseline, which may be supported by previous sympathetic nervous system overactivity data in BPS/IC patients. Sensorimotor network-decreased activation at full bladder status can also be justified by the brain’s cross-network connectivity, as described above.

Our pilot study has several limitations: the lack of a control group (the ethics committee did not approve patient setup with controls), a smaller number of enrolled patients, the impossibility to use a seed-based analysis or evaluating functional connectivity on resting-state functional brain MRI, and the absence of a quantitative method used to analyze functional brain MRI.

To increase external validity, functional brain MRI investigations analyzing images by quantitative methods and more extensive samples are desirable.

We hope that a correlation between severity scores/sympathetic nervous system activity of BPS/IC and network activation can be ultimately assessed soon. Despite the actual diagnostic and prognostic value of functional brain MRI in BPS/IC management is far to come. We believe this imaging tool can be extremely useful in the future in the study of central pain-related mechanisms, potential treatment mechanisms, and evaluating CNS potential changes following future treatments. Furthermore, the documentation of CNS changes is in favor of managing BPS/IC as an organic and serious condition, contrary to an organ approach, or even a psychologic condition, as it still is managed by some clinicians.

We concluded that both CSN pathways, DMN and SMN, seem to be actively involved in BPS/IC, as previously reported for chronic pain syndromes. Despite being early for its routine use, the role of functional neuroimaging in diagnosis, prognostic, and treatment-response evaluation should be seriously evaluated.

## Figures and Tables

**Table 1.** Patients’ Characteristics

Patient	Age	Gender	VAS	ESSIC	OSS	DFr	NFr	QoL	MVV
SAGC	45	Female	6	2C	24	10	2	5	300 mL
MLCM	49	Female	7	3B	25	6	2	4	300 mL
APPP	33	Female	6	3C	31	12	2	6	250 mL
FMMDS	44	Female	7	3C	23	8	2	4	300 mL
MRMC	37	Female	8	3C	28	10	4	6	200 mL
MICE	38	Female	7	3C	24	12	4	6	200 mL

**Table 2.** Results: A Qualitative Comparison

Patient	Default Mode Network Empty Bladder vs Full Bladder	Sensorimotor Network Empty Bladder vs Full Bladder
SAGC	Empty = Full	Empty > Full
MLCM	Empty = Full	Empty > Full
APPP	Empty = Full	Not possible to evaluate
FMMDS	Empty = Full	Empty > Full
MRMC	Empty = Full	Empty > Full
